# U.S. consumers’ processing of information about CRISPR-edited pork products

**DOI:** 10.1080/21645698.2026.2719351

**Published:** 2026-08-26

**Authors:** Joseph Opoku Gakpo, Bhavisha Gulabrai, Catherine E. Sanders, Jean A. Parrella, Joe Proudman, Trish Berger, Frank Mitloehner

**Affiliations:** a Department of Agricultural and Human Sciences, North Carolina State University, Raleigh, NC, USA; b Department of Agricultural, Leadership, and Community Education, Virginia Tech, Blacksburg, VA, USA; c Clarity and Leadership for Environmental Awareness and Research (CLEAR) Center, University of California, Davis, CA, USA

**Keywords:** CRISPR, evidence-based communication, gene editing, information processing, RISP model

## Abstract

The commercialization of CRISPR gene-edited pork is advancing rapidly, following the U.S. Food and Drug Administration’s approval of gene-edited pigs resistant to Porcine Reproductive and Respiratory Syndrome (PRRS). As these products move closer to market entry, understanding how consumers seek, process, and avoid information about them is critical for developing effective communication strategies. Guided by the Risk Information Seeking and Processing (RISP) model, this study examined factors influencing information seeking, information avoidance, and information processing related to CRISPR-edited pork products among U.S. consumers (*n* = 2,006). Results show higher information sufficiency thresholds were associated with greater information seeking and lower information avoidance. Information seeking was strongly and positively correlated with systematic processing. Relevant channel beliefs and perceived information gathering capacities were positively associated across communication channels, suggesting the need for integrated communication approaches. Relevant channel beliefs for news media and social media were positively associated with information seeking, while stronger relevant channel beliefs for Extension were associated with lower information seeking. Respondents with some college education reported higher information seeking than those with only a high school diploma or GED, while older adults and individuals with higher education levels reported lower information avoidance. Results also showed that respondents exhibited high intentions to seek information and low tendencies to avoid information, suggesting openness to learning about CRISPR-edited pork. Participants also reported engaging more in systematic processing than heuristic processing, indicating a preference for careful and analytical evaluation of information. Findings highlight the importance of audience segmentation, multi-channel communication strategies, and evidence-based messaging to support informed public engagement with CRISPR-edited food technologies.

## Introduction

The ways in which individuals search for and engage with information about risk are central to understanding how people respond to scientific and health-related issues.^1–3^ Existing research has shown that when people actively engage in seeking out information, they are often better positioned to adopt behaviors that reduce exposure to environmental and health risks.^2^ Risk Information Seeking and Processing (RISP) describes the cognitive and behavioral processes through which individuals look for, interpret, and use information when faced with uncertain or potentially harmful situations.^1,4^ This process is shaped by multiple psychological and contextual influences that determine both the motivation and capacity to engage with risk-related content.^4^ Seeking out information is effortful, meaning individuals are more likely to do so when they perceive clear value or necessity in the information for guiding their decisions.

The RISP framework developed by Griffin et al.^1^ suggests that individuals’ decisions to seek and process risk information are shaped by a range of personal, social, and situational factors, including their perceptions of the risk, emotional responses, information needs, confidence in their ability to understand information, and beliefs about the usefulness of communication channels. At the heart of the RISP framework is the idea that information seeking is driven by a perceived gap between what individuals already know and what they believe they need to know to adequately address a risk.^2^ When this perceived gap exists, individuals are motivated to reduce uncertainty by actively searching for additional information and engaging more deeply with available messages. The model suggests that individuals are more likely to engage in deliberate and analytical processing when they actively pursue information through multiple channels rather than relying on quick or heuristic judgments.^1^

Huang et al.^4^ state that the RISP model has been widely applied to study communication of infectious disease outbreaks and vaccination behavior in recent years. The model has also been used in assessing information seeking and processing habits on genetically modified organisms.^3,5^ Stakeholders involved in genetic engineering must pay close attention to how lay audiences process information in order to improve communication strategies and support informed public engagement.^3^

In this study, we extend the RISP framework to CRISPR-edited pork. Clustered regularly interspaced short palindromic repeats (CRISPR) is one of the leading gene-editing (GEd) technologies within the food system.^6–8^ Unlike earlier forms of genetic engineering, such as genetic modification (transgenics) that involved the random insertion of foreign DNA elements into the genomes of target organisms, current gene-edited organisms result from altering existing genes without using external genetic material, appropriating a naturally occurring process in bacteria.^6,8–11^ This distinction has led to expectations that the public will view GEd more favorably than transgenics,^6–8^ in part because there is likely no insertion of foreign genetic materials into non-host organisms.^8^ GEd’s affordability and technical simplicity also increase its accessibility, which may improve its public image and expand its adoption potential.^7^

The livestock industry has benefitted significantly from CRISPR research,^12^ from developing disease-resistant cattle, to improving fiber quality in goats and enhancing poultry growth rates and immunity.^13–15^ In the pork industry, researchers have used CRISPR to develop disease-resistant pigs.^16–18^ The U.S. Food and Drug Administration recently approved CRISPR GEd pigs resistant to Porcine Reproductive and Respiratory Syndrome (PRRS).^19^ The pork industry faces a major challenge from PRRS.^20–24^ PRRS virus causes reproductive problems such as stillbirths and weak piglets, as well as respiratory issues such as pneumonia. It can be transmitted through direct physical contact between pigs or via airborne infectious particles.^20,24,25^ PRRS is recognized as one of the most destructive diseases in pig farming.^20–22^ From 2016 to 2020, PRRS caused an estimated US$1.2 billion loss annually to the U.S. economy.^26^ There is high expectation that CRISPR-edited pigs will reduce losses and increase the sustainability of the pork industry.^16^ The ability to introduce PRRSV resistance in pigs through CRISPR/Cas9 gene editing provides an opportunity to improve swine health while significantly reducing production costs on farms,^16^ which could benefit U.S. consumers and the broader economy.

Despite these advancements, few studies have explored public perceptions and information behaviors related to gene-edited animals in the U.S., particularly for pigs. Existing work has largely focused on cattle,^27–29^ with findings showing that factors such as animal welfare framing, perceived benefits, social norms, and risk perceptions influence consumer attitudes and willingness to consume these products. Additionally, there is limited research investigating consumer perception of information channels and its impact on their information processing behaviors for risk-related information. Information channels often significantly influence the pathways of information dissemination, particularly within environmental and health communication contexts.^30,31^ Specifically, perception of information source credibility and/or trust can influence whether an individual exposes themselves to information from the source, pays attention to the source, and whether they will act on information presented by the source.^30^ Studies on other emerging food biotechnologies suggest that different communication channels, such as news media, social media, interpersonal networks, and Extension services, vary widely in perceived credibility, accessibility, and influence on public decision-making.^32,33^ Thus, information channel pathways related to consumer information processing is a key area of investigation needed to provide evidence-based science communication strategies for emerging agricultural technologies, such as CRISPR-edited pork.

## Theoretical Framework

**Figure 1.**
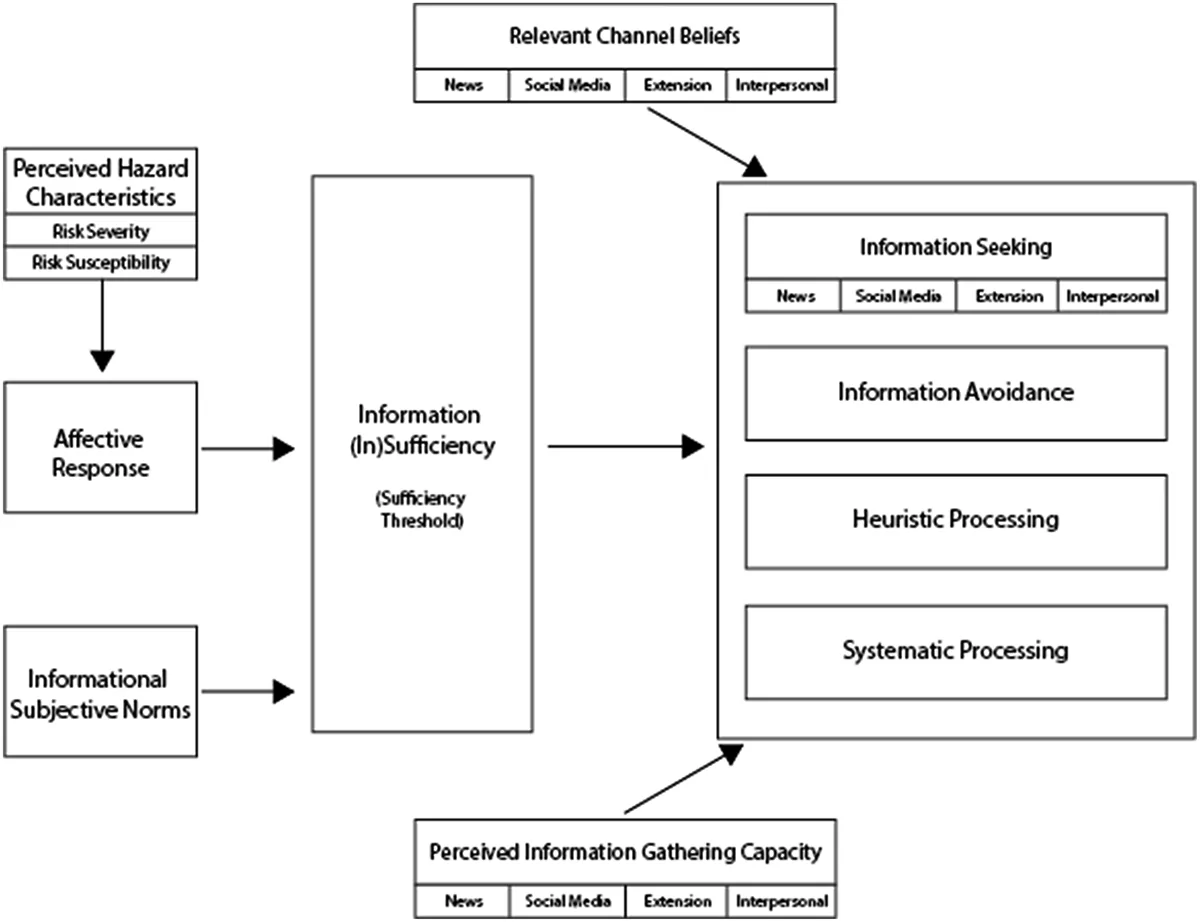
The risk information seeking and processing model (adapted from Hwang and Jeong^36^.

The Risk Information Seeking and Processing (RISP) model provides a comprehensive framework for understanding how people engage with information about potential risks,^1^ including those associated with emerging food technologies. Integrating elements of Eagly and Chaiken’s^34^ Heuristic-Systematic Model (HSM) and Ajzen’s^35^ Theory of Planned Behavior (TPB), RISP posits that an individual’s engagement with risk information is influenced by multiple interacting factors. These factors include: (1) subjective knowledge, which refers to an individual’s self-perceived understanding of a risk; (2) perceived hazardous characteristics, which involve an individual’s sense of the severity and susceptibility of the risk; (3) affective response, which refers to the emotional reactions such as worry or anxiety toward the risk; and (4) informational subjective norms, which reflect the social pressures that influence an individual’s decision to seek information.^36^ See Figure 1 for a visual representation of the RISP model.

Information insufficiency determines whether an individual feels they have enough information to make a decision.^33,37^ Additional factors include sufficiency threshold, which refers to the level of knowledge an individual believes is necessary to be adequately informed about the risk. Other variables include relevant channel beliefs (RCB), which pertain to an individual’s perceptions of the credibility and usefulness of various information sources. Adapted from Sanders et al.^38^, the current study operationalized information sources as news media, social media, Cooperative Extension (henceforth referred to as Extension), and interpersonal sources, which include family and friends. Perceived information-gathering capacity (PIGC) refers to the confidence an individual has in their ability to effectively gather and process relevant information.^33,36^ The current study measured PIGC with the same four information sources as RCB (news media, social media, Extension, and interpersonal). RCB and PIGC perceptions of various channels and information sources collectively shape whether individuals seek or avoid information, how deeply they process it (systematically versus heuristically), and how this ultimately impacts their behaviors.^1^ Importantly, they interact dynamically, influencing whether individuals engage with information in a more systematic manner - referring to deep cognitive engagement with presented information, or if they rely on heuristics - cognitive shortcuts and peripheral cues, to make decisions about presented information.^1^

The RISP framework has been applied to studies on health risks, food safety, and emerging technologies such as genetic modification (GM) and CRISPR GEd to provide valuable insights into public decision-making regarding emerging risks and technologies.^5,32,33^ Martinez et al.^33^ applied the RISP model to study how Generation Z (people born between 1997 and 2012) engages with CRISPR-related content on social media, revealing that individuals in this demographic often process risk information heuristically, relying on simple cues rather than in-depth analysis. Jennings^32^ also found that the source of risk information, whether personal or mediated by news outlets, can significantly influence individuals’ beliefs and behaviors regarding transgenics.

Recent work shows that channel-specific RCBs and PIGC can have distinct influences on behavior.^5,32,33^ The current study examines the impact of information insufficiency, sufficiency threshold, channel-specific RCBs, and channel-specific PIGCs on four outcome variables: 1) information seeking; 2) information avoidance; 3) systematic processing; and 4) heuristic processing (see the left-hand side of the model presented in Figure 1). This study extends scholarship related to the RISP framework to assess how U.S. consumers’ beliefs about and confidence in various channels shape their likelihood of seeking or avoiding information about CRISPR-edited pork, and whether they engage with it systematically or heuristically. This approach allows for a more nuanced understanding of communication dynamics and offers direct guidance for tailoring outreach strategies to maximize engagement and informed decision-making.

## Methods

This study applies the RISP model^1^ to examine how factors such as information sufficiency, sufficiency threshold, relevant channel beliefs, and perceived information gathering capacity across multiple channels relate to U.S. consumers’ information seeking, avoidance, and processing behaviors regarding CRISPR-edited pork. Two research questions guided the study:

1. What effects do information sufficiency, sufficiency threshold, relevant channels beliefs (for news media, social media, interpersonal, and Extension), and perceived information gathering capacity (for news media, social media, interpersonal, and Extension) have on respondents’ information seeking (M1), information avoidance (M2), systematic processing (M3), and heuristic processing (M4), while controlling for age, education, gender, and political ideology?
2. Is there a difference between respondents’ relevant channel beliefs, perceived information gathering capacity, and information seeking intentions when it comes to information about CRISPR-edited pork products from news media, social media, interpersonal, and Extension channels?

### Survey Development

We developed the survey instrument by adapting measures from various sources in the risk communication literature (see Table 1). In several cases, we combined or reduced items from previously published scales to retain those that align most directly with the conceptual definition of each construct, while also minimizing respondent fatigue. We used 7-point Likert scales ranging from strongly disagree (1) to strongly agree (7), with neither agree nor disagree as the midpoint. The only question that deviated from this format was information sufficiency threshold, for which we used a 0–10 sliding scale asking respondents to estimate how much knowledge they think they need to have about CRISPR-edited pork products (0 = I need to know nothing, 10 = I need to know everything). We used the sliding scale because the construct was intended to capture how much information respondents felt they needed, which differed conceptually from the other variables measured using agreement-based items. We recognize that the different scale format may limit direct comparison of coefficient magnitudes across variables; however, regression analyses do not require predictors to use the same response scales.

**Table 1.** Survey constructs, items, scales, and adapted sources.

Variable	Items	Scale	Source
Affective	1. I feel concerned about CRISPR-edited pork products. 2. I feel worried about CRISPR-edited pork products. 3. I feel anxious about CRISPR-edited pork products.	7-point Likert scale	Hwang and Jeong^36^; Yang et al.,^39^
Risk judgement – severity	1. The dangers of CRISPR-edited pork products are severe. 2. The dangers of CRISPR-edited pork products are serious. 3. The dangers of CRISPR-edited pork products are significant.	7-point Likert scale	Hwang and Jeong^36^; Yang et al.,^39^
Risk judgement – susceptibility	1. I am at risk of experiencing negative effects from CRISPR-edited pork products. 2. I am personally susceptible to potential problems related to CRISPR-edited pork products. 3. I am likely to be affected by any potential risks associated with CRISPR-edited pork products.	7-point Likert scale	Hwang and Jeong^36^; Yang et al.,^39^
Sufficiency threshold	Respondents estimated how much knowledge they think they need to have about CRISPR-edited pork products (0 = I need to know nothing, 10 = I need to know everything).	Sliding scale (0–10)	Hwang and Jeong^36^
Information avoidance	1. I avoid information about CRISPR-edited pork products. 2. I don’t want more information about CRISPR-edited pork products. 3. I refuse to listen to information about CRISPR-edited pork products.	7-point Likert scale	Hwang and Jeong^36^
Systematic processing	1. When I see or hear information about CRISPR-edited pork products, I am likely to stop and think about it. 2. When I encounter information about CRISPR-edited pork products, I make a strong effort to carefully examine the information. 3. If I need to make a decision about CRISPR-edited pork products (e.g., purchasing them), the more viewpoints I get the better.	7-point Likert scale	Hwang and Jeong^36^; Hingmann^40^; Griffin et al.^41^
Informational subjective norms	1. Others would expect me to seek information about CRISPR-edited pork products. 2. People who are important to me think that I should seek information about CRISPR-edited pork products. 3. My loved ones would want me to seek information about CRISPR-edited pork products.	7-point Likert scale	Hwang and Jeong^36^; Yang et al.^39^
Information seeking	1. I plan to seek information about CRISPR-edited pork products. 2. I will try to seek information about CRISPR-edited pork products. 3. I intend to find more information about CRISPR-edited pork products.	7-point Likert scale	Hwang and Jeong^36^
Heuristic processing	1. When I see or hear information about CRISPR-edited pork products, I rarely spend much time thinking about it. 2. When I encounter information about CRISPR-edited pork products, I focus on only a few key points. 3. If I need to make a decision about CRISPR-edited pork products (e.g., purchasing them), the advice of one expert is enough for me.	7-point Likert scale	Hwang and Jeong^36^; Trumbo^42^
Relevant channel beliefs	1. News media/Social media/People I know personally/Extension often exaggerate information about CRISPR-edited pork products. 2. News media/Social media/People I know personally/Extension often represent their own bias about CRISPR-edited pork products. 3. Information about CRISPR-edited pork products from news media/social media/people I know personally/Extension is not credible.	7-point Likert scale	Hwang and Jeong^36;39^ Researcher-developed
Perceived information gathering capacity	1. I could easily get all the information I need about CRISPR-edited pork products from news media/social media/people I know personally/Extension. 2. I could easily get useful information about CRISPR-edited pork products from news media/social media/people I know personally/Extension. 3. I know where to go to get information about CRISPR-edited pork products from news media/social media/people I know personally/Extension.	7-point Likert scale	Hwang and Jeong^36^; Researcher-developed
Information seeking intentions	1. I will try to find information about CRISPR-edited pork products from news media/social media/people I know personally/Extension in the near future. 2. I intend to find information about CRISPR-edited pork products from news media/social media/people I know personally/Extension in the near future. 3. I will use news media/social media/people I know personally/Extension to find more information about CRISPR-edited pork products in the near future.	7-point Likert scale	Hwang and Jeong^36;39^

To establish face validity of the instrument, we requested a review from two experts in agricultural and environmental science communication and one animal biotechnology specialist, each of whom provided suggestions to improve clarity and accuracy. To assess the construct validity of the adapted scales, we conducted exploratory factor analyses (EFA) and reliability analyses for each multi-item construct (see Table 2). We used principal axis factoring to extract factors and used the Kaiser–Meyer–Olkin (KMO) measure to assess sampling adequacy.^43^ We also used Bartlett’s Test of Sphericity to examine interrelationships among items and determine whether the data were suitable for factor analysis.^44^ The KMO values were acceptable for all constructs, ranging from 0.70 to 0.77, and Bartlett’s tests were significant for all constructs, indicating the data were factorable. Because only one factor emerged for each construct, rotation was unnecessary. All factor loadings exceeded the recommended cutoff value of 0.30,^45^ ranging from 0.71 to 0.96, and therefore supporting the unidimensionality of the adapted scales. Cronbach’s alpha coefficients indicated acceptable internal consistency reliability across constructs, ranging from 0.80 to 0.95.^46^

**Table 2.** Exploratory factor analysis and reliability results for scales.

Construct	α	KMO	Bartlett’s Test	Factor Loadings
Affective	0.91	0.75	*χ*^2^(3) = 4,257.78, *p* < .001	Item 1 = 0.92; Item 2 = 0.94; Item 3 = 0.91
Risk judgement – severity	0.91	0.76	*χ*^2^(3) = 4,119.74, *p* < .001	Item 1 = 0.92; Item 2 = 0.93; Item 3 = 0.91
Risk judgement – susceptibility	0.91	0.75	*χ*^2^(3) = 3,951.11, *p* < .001	Item 1 = 0.90; Item 2 = 0.93; Item 3 = 0.92
Information avoidance	0.92	0.76	*χ*^2^(3) = 4,617.48, *p* < .001	Item 1 = 0.92; Item 2 = 0.94; Item 3 = 0.94
Systematic processing	0.86	0.73	*χ*^2^(3) = 2,859.87, *p* < .001	Item 1 = 0.88; Item 2 = 0.91; Item 3 = 0.87
Informational subjective norms	0.90	0.75	*χ*^2^(3) = 3,836.46, *p* < .001	Item 1 = 0.90; Item 2 = 0.93; Item 3 = 0.92
Information seeking	0.94	0.77	*χ*^2^(3) = 5,365.64, *p* < .001	Item 1 = 0.94; Item 2 = 0.95; Item 3 = 0.94
Heuristic processing	0.80	0.70	*χ*^2^(3) = 1,941.03, *p* < .001	Item 1 = 0.71; Item 2 = 0.78; Item 3 = 0.69
Relevant channel beliefs – news media	0.86	0.72	*χ*^2^(3) = 2,730.87, *p* < .001	Item 1 = 0.87; Item 2 = 0.90; Item 3 = 0.87
Relevant channel beliefs – social media	0.87	0.73	*χ*^2^(3) = 3,017.21, *p* < .001	Item 1 = 0.90; Item 2 = 0.91; Item 3 = 0.87
Relevant channel beliefs – interpersonal	0.89	0.74	*χ*^2^(3) = 3,501.44, *p* < .001	Item 1 = 0.84; Item 2 = 0.86; Item 3 = 0.80
Relevant channel beliefs – Extension	0.88	0.74	*χ*^2^(3) = 3,260.31, *p* < .001	Item 1 = 0.83; Item 2 = 0.84; Item 3 = 0.80
Perceived information gathering capacity – news media	0.87	0.73	*χ*^2^(3) = 2,964.14, *p* < .001	Item 1 = 0.90; Item 2 = 0.91; Item 3 = 0.86
Perceived information gathering capacity – social media	0.91	0.75	*χ*^2^(3) = 4,060.53, *p* < .001	Item 1 = 0.85; Item 2 = 0.89; Item 3 = 0.84
Perceived information gathering capacity – interpersonal	0.94	0.77	*χ*^2^(3) = 5,127.05, *p* < .001	Item 1 = 0.94; Item 2 = 0.95; Item 3 = 0.94
Perceived information gathering capacity – Extension	0.87	0.73	*χ*^2^(3) = 3,004.45, *p* < .001	Item 1 = 0.90; Item 2 = 0.91; Item 3 = 0.87
Information seeking intentions – news media	0.93	0.76	*χ*^2^(3) = 4,847.48, *p* < .001	Item 1 = 0.94; Item 2 = 0.95; Item 3 = 0.93
Information seeking intentions – social media	0.95	0.77	*χ*^2^(3) = 6,326.80, *p* < .001	Item 1 = 0.96; Item 2 = 0.96; Item 3 = 0.95
Information seeking intentions – interpersonal	0.95	0.77	*χ*^2^(3) = 5,849.66, *p* < .001	Item 1 = 0.95; Item 2 = 0.96; Item 3 = 0.95
Information seeking intentions – Extension	0.94	0.77	*χ*^2^(3) = 5,156.21, *p* < .001	Item 1 = 0.94; Item 2 = 0.95; Item 3 = 0.94

Prior to respondents answering questions, we provided the following definition of CRISPR/Cas9 to ensure they considered gene editing within an agricultural context, as the technology has applications beyond food systems: “CRISPR/Cas9 (CRISPR for short) is a molecular tool used in agriculture to make precise genetic changes to crops and livestock. It is like using molecular scissors to edit the DNA of plants and animals used in food production, to improve their genetic traits.” This definition was adapted from Sanders et al.^38^ and Martinez.^33^ We intentionally used a brief, descriptive definition to provide respondents with baseline understanding of CRISPR; however, providing this information may have influenced respondents’ perceptions and responses.

Another important consideration is that not all respondents may have been familiar with the Cooperative Extension Service, which delivers research-based information to farmers and consumers in the United States.^47^ Prior to answering questions about Extension, we provided respondents with the following definition: “Cooperative Extension is a partnership of the federal government, states, and counties, that provides the populace with learning opportunities to benefit from research being undertaken in universities in the areas of agriculture, health, and human sciences. Cooperative Extension delivers research-based information from universities to farmers and communities to put knowledge into practice, improve food and nutritional security, improve livelihoods, and protect the environment.” We provided this definition to ensure respondents had baseline understanding of Extension prior to answering channel-specific questions.

### Data Collection

The North Carolina State University Institutional Review Board approved the study procedures prior to data collection. We conducted a non-probability sample using Qualtrics sampling services, and used quota sampling to reflect U.S. Census distributions for age, gender, education, income, and geographic region. We piloted the survey with a soft launch representing 1.4% of the sample size (*n* = 27) on October 8, 2024. We reviewed this data for discrepancies and data quality issues. We moved forward with final data collection between October 9 and October 18, 2024. We added an attention check question midpoint. Qualtrics representatives scrubbed the data and performed various quality checks, ensuring the validity of each response. They removed 115 poor responses from the original ones received.

### Sample

All respondents resided in the United States. Most of them identified as females, resided in the southern US, and were White. The largest proportions of respondents were between ages 25–34 and 35–44, earned between $25,000 and $49,999 annually, had completed high school or GED, lived in suburban areas (population between 50,000 and 249,999), and identified as politically moderate. Table 3 presents details on the socio-demographic characteristics of participants.

**Table 3.** Socio-demographic characteristics of participants (*n* = 2,006).

Characteristics	*f*	%
Gender		
Female	1,037	51.69
Male	953	47.51
Non-binary/third gender	11	0.55
Other	3	0.15
Prefer not to say	2	0.10
Age		
Below 18	2	0.10
18–24 y	154	7.68
25–34 y	421	20.99
35–44 y	385	19.19
45–54 y	271	13.51
55–64 y	292	14.56
65–74 y	317	15.80
75–84 y	144	7.18
85+ y	20	1.00
Ethnicity		
White	1,372	68.39
Black or African American	404	20.14
Hispanic, Latino/a/x, or Chicano/a/x	118	5.88
Asian or Asian American	57	2.84
Multiracial	38	1.89
American Indian or Alaska Native	16	0.80
Other	1	0.05
Income		
Less than $25,000	557	27.77
$25,000–$49,999	584	29.11
$50,000–$74,999	383	19.09
$75,000–$149,999	359	17.90
$150,000–$249,999	101	5.03
$250,000 or more	22	1.10
Highest Level of Education Completed		
High school or GED	654	32.60
Some college	466	23.23
Bachelor’s degree	378	18.84
Associates degree	238	11.86
Graduate degree (master’s, doctorate)	195	9.72
Some high school	75	3.74
Political Ideology		
Moderate	819	40.83
Conservative	356	17.75
Liberal	319	15.90
Very conservative	251	12.51
Very liberal	222	11.07
Apolitical	39	1.94
Urban/Rural		
Suburban (population between 50,000 and 249,999)	824	41.08
Urban (population of 250,000 or more)	666	33.20
Rural (population less than 50,000)	516	25.72
Region		
South	779	38.83
West	447	22.28
Midwest	431	21.49
Northeast	349	17.40

### Data Analysis

We used Stata/SE 18.0 to analyze the data, which contained no missing values. We conducted simultaneous multiple linear regression analyses and dummy coded the categorical variables, using the group in each category with the largest sample size as the reference group. The goal of the regression analyses was not to maximize explained variance but to examine the relative influence of information sufficiency, sufficiency threshold, and channel-specific perceptions (i.e., relevant channel beliefs, and perceived information gathering capacity across news media, social media, interpersonal sources, and Extension) on the four outcome variables: information seeking (M1), information avoidance (M2), systematic processing (M3), and heuristic processing (M4). The proposed relationships between predictor and outcome variables can be seen in Fig. 1. This approach allowed for a comparative assessment of how different communication channels shape participants’ information seeking and avoidance tendencies, as well as their information processing behaviors in the context of CRISPR-edited pork products. Rather than aggregating across channels or modeling each channel separately, we included all channel-specific predictors simultaneously to evaluate their unique contributions.

We first assessed the assumptions of linearity, normality, homoscedasticity, and multicollinearity for each model. To assess linearity, we used residual vs. predictor plots, which showed the Lowess lines closely aligned with the linear reference lines for most of the distribution. To assess normality, we used density plots and QQ plots, which showed the residuals approximately normally distributed for each model. To assess homoscedasticity, we used residual vs. fitted plots, which showed that the variance of residuals was consistent across fitted values. We assessed multicollinearity using variance inflation factor (VIF) values. All predictors had VIFs ranging from 1.10 to 2.59, and a mean VIF of 1.50, indicating low multicollinearity. Because the study relied on self-reported survey data collected at a single point in time, we assessed the potential for common method variance using Harman’s single-factor test. The unrotated factor solution showed that the first factor accounted for 33.04% of the variance, suggesting common method variance was unlikely to substantially influence the results.

## Results

Respondents reported neutral information sufficiency on CRISPR-edited pork but a high sufficiency threshold (Table 4). They reported somewhat high information seeking and somewhat low information avoidance. Respondents engaged in relatively higher levels of systematic processing, compared to heuristic processing. Social media recorded the highest relevant channel beliefs, followed by news media, then Extension and interpersonal. Extension recorded the highest perceived information gathering capacity, followed by news media, social media, then interpersonal.

**Table 4.** Means, standard deviations, and Cronbach’s alpha coefficients of variables (*n* = 2,006).

Variables	*M*	*SD*	α
Information sufficiency	3.81	1.71	0.91
Sufficiency threshold (10-point scale)	6.78	2.83	–
Information seeking	4.88	1.55	0.92
Information avoidance	3.33	1.75	0.92
Systematic processing	4.96	1.34	0.83
Heuristic processing	3.99	1.46	0.78
Relevant Channel Beliefs	–	–	–
News media	4.54	1.33	0.84
Social media	4.73	1.35	0.86
Interpersonal	3.99	1.54	0.88
Extension	4.18	1.34	0.86
Perceived information gathering capacity	–	–	–
News media	3.89	1.55	0.86
Social media	3.80	1.70	0.89
Interpersonal	3.64	1.76	0.92
Extension	4.45	1.33	0.85
Information seeking intentions	4.88	1.55	0.94

Note. 1.00–1.49 = strongly disagree; 1.50–2.49 = disagree; 2.50–3.49 = somewhat disagree; 3.50–4.49 = neutral; 4.50–5.49 = somewhat agree; 5.50–6.49 = agree; and 6.50–7.00 = strongly agree.

We used Cohen’s^48^ guidelines for interpreting the magnitude of Pearson correlation coefficients. Correlations between psychological variables ranged from negligible to very strong in magnitude (Table 5). For example, information seeking and heuristic processing were negligibly correlated (*r* = 0.04, *p* = .070), as were heuristic and systematic processing (*r* = 0.04, *p* = .077). Moderate correlations were observed between information sufficiency and information avoidance (*r* = 0.42, *p* < .001), as well as between systematic processing and sufficiency threshold (*r* = 0.43, *p* < .001). A very strong correlation existed between information seeking and systematic processing (*r* = 0.71, *p* < .001).

**Table 5.** Pearson r correlations among psychological variables (*n* = 2,006).

Psychological Variables	1	2	3	4	5	6
Information sufficiency (1)	1					
Sufficiency threshold (2)	0.15***	1				
Information avoidance (3)	0.42***	−0.06*	1			
Information seeking (4)	0.34***	0.42***	−0.13***	1		
Systematic processing (5)	0.27***	0.43***	−0.14***	0.71***	1	
Heuristic processing (6)	0.55***	0.01	0.64***	0.04	0.04	1

Note. *p < .05; **p < 0.01; ***p < .001.

Correlations between channel-specific variables ranged from moderate to substantial in strength (Table 6). Relevant channel beliefs across news media, social media, interpersonal, and Extension were positively associated, with the most substantial correlation between beliefs about news media and social media (*r* = 0.59, *p* < .001). Perceived information gathering capacity variables were also strongly related, particularly between perceptions of social media and interpersonal sources (*r* = 0.66, *p* < .001), and between news media and interpersonal sources (*r* = 0.65, *p* < .001). Correlations between relevant channel beliefs and perceived information gathering capacity within the same channel were mostly moderate. For example, relevant channel beliefs about news media were moderately correlated with perceived information gathering capacity from news media (*r* = 0.30, *p* < .001).

**Table 6.** Pearson r correlations among channel-specific variables (*n* = 2,006).

Channel-Specific Variables	1	2	3	4	5	6	7	8
News media RCB (1)	1							
Social media RCB (2)	0.59***	1						
Interpersonal RCB (3)	0.51***	0.47***	1					
Extension RCB (4)	0.48***	0.37***	0.49***	1				
News media PIGC (5)	0.30***	0.26***	0.48***	0.38***	1			
Social media PIGC (6)	0.32***	0.16***	0.47***	0.43***	0.69***	1		
Interpersonal PIGC (7)	0.38***	0.27***	0.55***	0.48***	0.65***	0.66***	1	
Extension PIGC (8)	0.29***	0.32***	0.40***	0.28***	0.50***	0.44***	0.49***	1

Note. RCB = Relevant channel beliefs; PIGC = Perceived information gathering capacity. *p < .05; **p < .01; ***p < .001.

The regression model (M1), which included information sufficiency, sufficiency threshold, relevant channels beliefs (for news media, social media, interpersonal, and Extension), perceived information gathering capacity (for news media, social media, interpersonal, and Extension), and demographic controls (age, gender, education, and political ideology) accounted for 31.95% of the variance in information seeking (adjusted R^2^ = 30.84%; see Table 7). The model was statistically significant (*F*(32, 1973) = 28.94, *p* < .001).

**Table 7.** Results from the regression models with information seeking and information avoidance as the dependent variables (*n* = 2,006).

Predictors	Information Seeking (M1)				Information Avoidance (M2)			
	*B* (S.E.)	*t*	*p*	*β*	*B* (S.E.)	*t*	*p*	*β*
Intercept	1.35 (.17)	7.88	<0.001	–	0.82 (.18)	4.66	<0.001	–
Information sufficiency	0.14 (.02)	5.88	<0.001	0.16	0.04 (.02)	1.49	0.137	0.04
Sufficiency threshold	0.18 (.01)	16.48	<0.001	0.32	−0.09 (.01)	−8.40	<0.001	−0.15
News media RCB	0.10 (.03)	3.26	0.001	0.08	0.16 (.03)	5.00	<0.001	0.12
Social media RCB	0.09 (.03)	3.09	0.002	0.08	−0.08 (.03)	−2.89	0.004	−0.07
Interpersonal RCB	0.03 (.03)	0.96	0.335	0.03	0.14 (.03)	5.12	<0.001	0.12
Extension RCB	−0.06 (.03)	−2.14	0.033	−0.05	0.28 (.03)	10.08	<0.001	0.22
News media PIGC	−0.04 (.03)	−1.47	0.141	−0.04	0.28 (.03)	9.32	<0.001	0.25
Social media PIGC	0.06 (.03)	2.32	0.021	0.07	0.08 (.03)	2.87	0.004	0.08
Interpersonal PIGC	0.02 (.03)	0.61	0.543	0.02	0.05 (.03)	1.97	0.049	0.05
Extension PIGC	0.20 (.03)	7.41	<0.001	0.17	−0.09 (.03)	−3.06	0.002	−0.07
Age	–	–	–	–	–	–	–	–
Below 18	−0.47 (.92)	−0.51	0.610	−0.30	0.27 (.95)	0.29	0.773	0.16
18–24 y	0.10 (.12)	0.80	0.421	0.06	0.22 (.13)	1.76	0.078	0.13
35–44 y	0.00 (.09)	0.04	0.971	0.00	0.11 (.10)	1.11	0.267	0.06
45–54 y	0.05 (.11)	0.52	0.603	0.04	−0.16 (.11)	−1.48	0.138	−0.09
55–64 y	0.15 (.11)	1.38	0.169	0.10	−0.20 (.11)	−1.80	0.072	−0.11
65–74 y	0.14 (.11)	1.29	0.199	0.09	−0.44 (.11)	−4.01	<0.001	−0.25
75–84 y	−0.16 (.14)	−1.16	0.245	−0.10	−0.17 (.14)	−1.22	0.224	−0.10
85+ y	0.33 (.30)	1.09	0.276	0.21	−0.91 (.31)	−2.96	0.003	−0.52
Gender	–	–	–	–	–	–	–	–
Male	−0.11 (.07)	−1.62	0.105	−0.07	−0.10 (.07)	−1.49	0.137	−0.06
Non-binary/third gender	−0.68 (.40)	−1.70	0.089	−0.44	0.15 (.41)	0.36	0.717	0.08
Other	−0.66 (.75)	−0.87	0.383	−0.42	−0.64 (.78)	−0.83	0.407	−0.37
Prefer not to say	0.78 (.92)	0.85	0.398	0.50	−0.65 (.95)	−0.69	0.491	−0.37
Education	–	–	–	–	–	–	–	–
Some high school	−0.01 (.16)	−0.03	0.973	−0.00	−0.01 (.16)	−0.03	0.975	−0.00
Some college	0.18 (.08)	2.32	0.021	0.12	−0.20 (.08)	−2.49	0.013	−0.12
Associates degree	0.03 (.10)	0.35	0.729	0.02	−0.26 (.10)	−2.55	0.011	−0.15
Bachelor’s degree	0.06 (.09)	0.75	0.454	0.04	−0.27 (.09)	−3.11	0.002	−0.16
Graduate degree	0.12 (.11)	1.12	0.263	0.08	−0.05 (.11)	−0.42	0.673	−0.03
Political ideology	–	–	–	–	–	–	–	–
Very liberal	0.07 (.10)	0.74	0.461	0.05	0.15 (.10)	1.46	0.144	0.09
Liberal	0.10 (.09)	1.16	0.248	0.06	−0.02 (.09)	−0.25	0.802	−0.01
Conservative	−0.10 (.08)	−1.23	0.217	−0.07	0.07 (.09)	0.87	0.385	0.04
Very conservative	−0.14 (.09)	−1.43	0.152	−0.09	0.08 (.10)	0.79	0.432	0.04
Apolitical	−0.22 (.21)	−1.04	0.299	−0.14	−0.13 (.22)	−0.60	0.550	−0.03

Note. RCB = Relevant channel beliefs; PIGC = Perceived information gathering capacity.

Holding all other variables constant, each additional point in information sufficiency was associated with an average increase of 0.14 in information seeking (*t*(1973) = 5.88, *p* < .001), while each additional point in information sufficiency threshold was associated with an average increase of 0.18 (*t*(1973) = 16.48, *p* < .001). Relevant channel beliefs for news media and social media were also positively associated with information seeking, with each additional point associated with average increases of 0.10 (*t*(1973) = 3.26, *p* = .001) and 0.09 (*t*(1973) = 3.09, *p* = .002), respectively. In contrast, each additional point in relevant channel beliefs for Extension was associated with an average decrease of 0.06 in information seeking (*t*(1973) = −2.14, *p* = .033). Additionally, each additional point in perceived information gathering capacity for social media and Extension was associated with average increases of 0.06 (*t*(1973) = 3.32, *p* = .021) and 0.20 (*t*(1973) = 7.41, *p* < .001) in information seeking, respectively. Regarding education, respondents with some college education had statistically significantly higher mean information seeking scores compared to respondents with a high school diploma or GED (*t*(1973) = 2.32, *p* = .021).

M2 included the same predictors as M1 but with information avoidance as the dependent variable and accounted for 43.26% of the variance (adjusted R^2^ = 42.34%; see Table 7). The model was statistically significant (*F*(32, 1973) = 47.01, *p* < .001). Holding all other variables constant, each additional point in information sufficiency threshold was associated with an average decrease of 0.09 in information avoidance (*t*(1973) = −8.40, *p* < .001). Relevant channel beliefs for news media, interpersonal, and Extension were positively associated with information avoidance, with each additional point associated with average increases of 0.16 (*t*(1973) = 5.00, *p* < .001), 0.14 (*t*(1973) = 5.12, *p* < .001), and 0.28 (*t*(1973) = 10.08, *p* < .001), respectively. In contrast, each additional point in relevant channel beliefs for social media was associated with an average decrease of 0.08 in information avoidance (*t*(1973) = −2.89, *p* = .004). Also, each additional point in perceived information gathering capacity for news media, social media, and interpersonal was associated with average increases of 0.28 (t(1973) = 9.32, *p* < .001), 0.08 (*t*(1973) = 2.87, *p* = .004), and 0.05 (*t*(1973) = 1.97, *p* = .049) in information avoidance, respectively. In contrast, each additional point in perceived information gathering capacity for Extension was associated with an average decrease of 0.05 in information avoidance (*t*(1973) = −3.06, *p* = .002). Regarding age, respondents aged 65–74 and 85 or older had statistically significantly lower mean information avoidance scores compared to respondents aged 25–35 (*t*(1973) = −4.01, *p* < .001; *t*(1973) = −2.96, *p* = .003), respectively. For education, respondents with some college, an associate’s degree, and a bachelor’s degree had statistically significantly lower mean information avoidance scores compared to respondents with a high school diploma or GED (*t*(1973) = −2.49, *p* = .013; *t*(1973) = −2.55, *p* = .011; *t*(1973) = −3.11, *p* = .002), respectively.

M3 included the same predictors as M1 and M2 but with systematic processing as the dependent variable and accounted for 35.33% of the variance (adjusted R^2^ = 34.28%; see Table 8). The model was statistically significant (*F*(32, 1973) = 33.68, *p* < .001). Holding all other variables constant, each additional point in information sufficiency and information sufficiency threshold was associated with average increases of 0.08 (*t*(1973) = 4.06, *p* < .001) and 0.15 (*t*(1973) = 16.73, *p* < .001) in systematic processing, respectively. Relevant channel beliefs for news media and social media were also positively associated with systematic processing, with each additional point associated with average increases of 0.13 (*t*(1973) = 4.94, *p* < .001) and 0.14 (*t*(1973) = 5.86, *p* < .001), respectively. In contrast, each additional point in relevant channel beliefs for Extension was associated with an average decrease of 0.06 in systematic processing (*t*(1973) = −2.62, *p* = .009). Also, each additional point in perceived information gathering capacity for Extension was associated with an average increase of 0.21 in systematic processing (*t*(1973) = 8.90, *p* < .001). Regarding age, respondents aged 55–64, 65–74, and 85 or older had statistically significantly higher mean systematic processing scores compared to respondents aged 25–35 (*t*(1973) = 2.41, *p* = .016; *t*(1973) = 4.11, *p* < .001; *t*(1973) = 3.22, *p* = .001), respectively. Respondents with some college education (*t*(1973) = 3.79, *p* < .001), a bachelor’s degree (*t*(1973) = 2.40, *p* = .016), and a graduate degree (*t*(1973) = 3.64, *p* < .001) also had statistically significantly higher mean systematic processing scores compared to respondents with a high school diploma or GED. Liberal respondents also had statistically significantly higher mean systematic processing scores compared to moderate respondents (*t*(1973) = 2.11, *p* = .035); however, very liberal respondents demonstrated no statistically significant difference in mean systematic processing scores.

**Table 8.** Results from the regression models with systematic processing and heuristic processing as the dependent variables (*n* = 2,006).

Predictors	Systematic Processing (M3)				Heuristic Processing (M4)			
	*B* (S.E.)	*t*	*p*	*β*	*B* (S.E.)	*t*	*p*	*β*
Intercept	1.46 (.14)	10.10	<0.001	–	0.67 (.14)	4.82	<0.001	–
Information sufficiency	0.08 (.02)	4.06	<0.001	0.11	−0.00 (.02)	−0.16	0.872	−0.00
Sufficiency threshold	0.15 (.01)	16.73	<0.001	0.32	−0.06 (.01)	−7.17	<0.001	−0.12
News media RCB	0.13 (.03)	4.94	<0.001	0.12	0.20 (.02)	8.26	<0.001	0.19
Social media RCB	0.14 (.02)	5.86	<0.001	0.14	0.01 (.02)	0.64	0.524	0.01
Interpersonal RCB	−0.01 (.02)	−0.57	0.571	−0.01	0.05 (.02)	2.16	0.031	0.05
Extension RCB	−0.06 (.02)	−2.62	0.009	−0.06	0.18 (.02)	8.07	<0.001	0.17
News media PIGC	0.04 (.02)	1.47	0.141	0.04	0.30 (.02)	12.57	<0.001	0.32
Social media PIGC	−0.01 (.02)	−0.24	0.810	−0.01	0.13 (.02)	5.72	<0.001	0.15
Interpersonal PIGC	−0.02 (.02)	−0.75	0.451	−0.02	0.03 (.02)	1.19	0.235	0.03
Extension PIGC	0.21 (.02)	8.90	<0.001	0.20	−0.00 (.02)	−0.03	0.977	−0.00
Age	–	–	–	–	–	–	–	–
Below 18	−1.00 (.78)	−1.28	0.201	−0.74	−0.42 (.75)	−0.55	0.582	−0.28
18–24 y	−0.02 (.10)	−0.22	0.827	−0.02	0.10 (.10)	1.04	0.298	0.07
35–44 y	0.02 (.10)	0.25	0.801	0.02	0.10 (.08)	1.30	0.193	0.07
45–54 y	0.11 (.09)	1.23	0.219	0.08	−0.02 (.09)	−0.21	0.837	−0.01
55–64 y	0.22 (.09)	2.41	0.016	0.16	0.04 (.09)	0.47	0.637	0.03
65–74 y	0.37 (.09)	4.11	<0.001	0.28	0.04 (.09)	0.45	0.649	0.03
75–84 y	0.17 (.11)	1.45	0.147	0.12	0.10 (.11)	0.92	0.360	0.07
85+ y	0.82 (.25)	3.22	0.001	0.61	0.09 (.24)	0.36	0.719	0.06
Gender	–	–	–	–	–	–	–	–
Male	−0.07 (.06)	−1.35	0.177	−0.06	0.12 (.05)	2.34	0.019	0.09
Non-binary/third gender	−0.65 (.34)	−1.92	0.054	−0.48	−0.13 (.32)	−0.40	0.687	−0.09
Other	−0.66 (.64)	−1.03	0.304	−0.49	0.42 (.61)	0.69	0.489	0.29
Prefer not to say	0.25 (.78)	0.32	0.751	0.18	−1.28 (.95)	−1.72	0.086	−0.89
Education	–	–	–	–	–	–	–	–
Some high school	−0.09 (.13)	−0.70	0.484	−0.07	0.12 (.13)	0.91	0.365	0.08
Some college	0.25 (.07)	3.79	<0.001	0.19	−0.06 (.06)	−0.99	0.324	−0.04
Associates degree	0.10 (.08)	1.20	0.230	0.08	0.02 (.08)	0.24	0.809	0.01
Bachelor’s degree	0.17 (.07)	2.40	0.016	0.13	0.05 (.07)	0.75	0.452	0.04
Graduate degree	0.34 (.09)	3.64	<0.001	0.25	0.06 (.09)	0.67	0.506	0.04
Political ideology	–	–	–	–	–	–	–	–
Very liberal	0.01 (.09)	0.06	0.952	0.00	0.16 (.08)	1.99	0.046	0.11
Liberal	0.15 (.07)	2.11	0.035	0.16	−0.04 (.07)	−0.61	0.541	−0.03
Conservative	−0.02 (.07)	−0.22	0.828	−0.01	−0.09 (.07)	−1.37	0.172	−0.06
Very conservative	−0.04 (.08)	−0.52	0.604	−0.03	−0.14 (.08)	−1.86	0.063	−0.10
Apolitical	0.16 (.18)	0.87	0.385	0.12	−0.01 (.17)	−0.07	0.947	−0.01

Note. RCB = Relevant channel beliefs; PIGC = Perceived information gathering capacity.

M4 included the same predictors as M1, M2, and M3 but with heuristic processing as the dependent variable and accounted for 48.86% of the variance (adjusted R^2^ = 48.03%; see Table 8). The model was statistically significant (*F*(32, 1973) = 58.91, *p* < .001). Holding all other variables constant, each additional point in information sufficiency threshold was associated with an average decrease of 0.06 in heuristic processing (*t*(1973) = −7.17, *p* < .001). Relevant channel beliefs for news media, interpersonal, and Extension were positively associated with heuristic processing, with each additional point associated with average increases of 0.20 (*t*(1973) = 8.26, *p* < .001), 0.05 (*t*(1973) = 2.16, *p* = .031), and 0.18 (*t*(1973) = 8.07, *p* < .001), respectively. Also, each additional point in perceived information gathering capacity for news media and social media was associated with average increases of .30 (*t*(1973) = 12.57, *p* < .001) and .13 (*t*(1973) = 5.72, *p* < .001) in heuristic processing, respectively. Lastly, male respondents had statistically significantly higher mean heuristic processing scores compared to female respondents (*t*(1973) = 2.34, *p* = .019), as did very liberal respondents compared to moderate respondents (*t*(1973) = 1.99, *p* = .046).

## Discussion

The findings of this current study show higher information sufficiency and higher sufficiency thresholds were associated with greater information seeking and lower information avoidance. This is consistent with studies that have reported a positive relationship between sufficiency threshold and information-seeking behavior when current knowledge is controlled for.^39,49–51^ However, findings from literature remain mixed, as other studies have found no significant effect of sufficiency threshold on information seeking.^52–54^ Higher information sufficiency and sufficiency thresholds increased systematic processing and were associated with lower heuristic processing. This finding is consistent with arguments by Chaiken et al.^55^ and Dunwoody and Griffin,^56^ who note that individuals are usually willing to expend the effort required to attain a level of confidence they consider adequate for making a decision (sufficiency threshold). When the threshold is low, individuals are more likely to rely on simple decision cues or heuristics, whereas a higher threshold encourages more extensive information seeking and careful evaluation (systematic processing).

Higher relevant channel beliefs (RCBs) for news media and social media were positively associated with information seeking, while higher RCBs for Extension was associated with a decrease in information seeking. RCBs reflect how much perceived bias different channels have and so a higher score implies respondents perceived that channel as more biased (or less credible). Individuals develop perceptions about the credibility, quality, and potential bias of information channels over time, and these beliefs influence how they seek, evaluate, and process information.^56,57^ The above results imply that respondents who perceived news media and social media as more biased tended to seek out more information, possibly to verify claims or obtain additional perspectives. This agrees with analysis by Kosicki and McLeod^57^ and Griffin et al.^1^ that individuals who view media as biased, overly influential, or of low quality are more likely to engage in active and critical evaluation of information, possibly as a way to guard against manipulation.

Meanwhile, perceiving Extension sources as more biased was associated with reduced information seeking, suggesting that diminished confidence in Extension may discourage engagement with CRISPR-edited pork information altogether. Also, higher RCBs about news media and social media promoted systematic processing, while higher RCBs about Extension slightly reduced systematic processing and increased heuristic processing. This suggests that skepticism toward news media and social media may motivate individuals to scrutinize information more carefully, whereas skepticism toward Extension sources may reduce trust in the information provided and encourage less effortful, heuristic processing. Extension has long been regarded as a trusted and go-to source of agricultural information,^58,59^ and in the context of emerging technologies such as CRISPR-edited pork, this trusted role becomes even more critical. To remain credible and effective, extension systems must be adequately equipped with up-to-date scientific knowledge, communication capacity, and institutional support to engage meaningfully on these innovations.

Greater perceived information gathering capacity (PIGC) through social media and Extension increased information seeking. Meanwhile, PIGC through news media, social media, and interpersonal channels increased information avoidance, whereas higher Extension capacity reduced avoidance. Greater PIGC through Extension strongly increased systematic processing. Meanwhile, greater PIGC through news media and social media strongly increased heuristic processing. This suggests that the influence of PIGC may vary across information channels and contexts. Dunwoody & Griffin^56^ found similar mixed relationships have been observed in previous studies, with some research finding that greater PIGC promotes information seeking and systematic processing, while other studies have reported the opposite effects, particularly for information avoidance and heuristic processing.

### Open to Learning About CRISPR-Edited Pork

Respondents reported being open to learning about CRISPR-edited pork, exhibiting a low tendency for information avoidance and a relatively high intention to seek information. This suggests that scientists and communicators could actively engage the public on this product, rather than shying away from engagement, a pattern seen with other biotechnology innovations in the past.^60,61^ To facilitate this engagement, communicators can leverage various channels, including science journalism through traditional media, social media, and other digital platforms, as well as practical, in-person engagements such as science talks and public outreach activities^62^^63,64^ Given the observed tendencies toward information seeking and low avoidance, continuous engagement could be particularly effective in building awareness, understanding, and trust.

Respondents also expressed neutral feelings about the amount of information they felt they needed on CRISPR-edited pork. However, they reported a high threshold for feeling adequately informed, suggesting they require a significant amount of information before reaching a level of comfort with the topic. While this indicates a need for more information, communicators should be cautious of information overload. We recommend applying a modular communication approach for CRISPR-edited pork information dissemination, starting with simple explanations and building toward more technical details for those who desire them.^65,66^ This method, which organizes messages into distinct, self-contained units, offers flexibility and allows audiences to progressively engage with content at their own pace by focusing on one unit and completing it before moving to the next. This approach can be applied to various types of content, such as videos, articles, and social media posts, by providing foundational information with the option to explore more detailed content based on individual interest. Creating a digital portal with videos, stories, and FAQs could be an effective way to implement this strategy.

The study’s results also revealed that respondents engaged in higher levels of systematic processing than heuristic processing. Given the prevalence of systematic processing, communicators should avoid oversimplifying messages about CRISPR-edited pork and instead support content with scientific evidence, data visualizations, and citations.^67–69^ Additionally, the prevalence of higher levels of systematic processing than heuristic processing supports the need for deep cultural tailoring of messages over surface-level tailoring. As explained by Zhou et al.,^70^ deep tailoring incorporates an audience’s underlying values, beliefs, and social norms, prompting more thoughtful systematic processing, while surface-level tailoring tends to serve as a quick cue for heuristic processors. The findings of this study, therefore, emphasize the need to tailor CRISPR pork information to address the psychographics (values, beliefs, and social norms) of specific audiences.

The findings also provide important insights into the channels respondents use to seek information. Relevant channel beliefs were highest for social media, followed by news media. RCBs were lowest for interpersonal sources, followed by extension. This means social media and news media have more negative public perceptions and interpersonal is the most positively perceived, with Extension being the second most positively perceived. However, just because social and news media have a negative perception does not mean people will avoid the information; but they may more critically engage with it. Meanwhile, perceived information gathering capacity was highest for Extension, followed by news media, social media, and interpersonal. This suggests that consumers perceive more established, and perhaps more trusted sources such as Extension as being the most capable of providing them with CRISPR-edited pork information. This aligns with previous research that suggests agricultural advisors and Extension agents have traditionally been trusted and authoritative sources of information and practical knowledge on sustainable agricultural practices.^71,72^

Information seeking was very strongly and positively correlated with systematic processing, suggesting that individuals who actively seek information are also more likely to process it analytically and carefully. Relevant channel beliefs across news media, social media, interpersonal, and Extension channels were positively associated. This means that respondents who viewed one communication channel positively were also likely to view the other channels positively. Perceived information gathering capacity variables were also strongly interrelated, indicating that respondents who felt confident gathering information from one source often felt similarly confident using other channels. The above emphasize the need for holistic communication strategies that harmonizes and complements messages across multiple platforms and channels to maximize audience engagement and information uptake.^5,73–75^

### How Demographics Impact Information Seeking and Processing

Respondents with some college education reported higher information seeking than those with only a high school diploma/GED. Meanwhile, older adults (65–74 and 85+) and respondents with higher education levels reported lower information avoidance. Older respondents, those with higher education, and liberals (but not identifying as “very liberal”) reported higher levels of systematic processing. Male and very liberal respondents reported higher heuristic processing scores than female and moderate respondents, respectively. Results demonstrate differences between the way self-identified liberals process information compared to individuals identifying as “very liberal.” This difference was observed but the reasons for this distinction are not immediately clear. The authors recommend further research to identify correlational or causal mechanisms into this distinction. One potential explanation could be that those more deeply entrenched in a political ideology (very liberal versus liberal), may rely on heuristic cues for information processing as they quickly determine whether this information supports or negates their preexisting beliefs. Thus, perhaps exploring the relationship between more extreme political ideologies and differences in informational processing behavior could elucidate these differences.

The differences in information seeking and processing emphasizes the need for audience segmentation so each group could be targeted using different channels and tailored messaging. Audience segmentation helps improve message effectiveness by allowing communicators to tailor content to the specific needs and preferences of different groups using language and channels that appeal to them.^76,77^ People above 55 y mainly rely on newspapers, radio, and television for their news information; those within the ages 35 to 54 usually use digital and traditional media alike; and those from 18 to 34 mainly rely on social media platforms.^78^ Communicators disseminating information about CRISPR-edited pork could adopt a segmented communication strategy where for older audiences, trusted traditional media (TV, print) is used to offer clear, simplified materials that explain the technology. For younger and/or more educated audiences, they can be targeted with explainer videos disseminated on social media. Martinez et al.^33^ found that Generation Z (people born between 1997 and 2012) often relied on simplified visual cues, such as animated infographics, to process information about CRISPR technology in food. Holt et al.^79^ also found that animated infographics improved recall of genetic engineering information, suggesting that more engaging visual formats may aid in the systematic processing of such complex topics. Communicators can consider a broad, multi-channel approach that supports complex, multi-layered engagements across various platforms.^80,81^

### Limitations of Study

CRISPR-edited pork is not yet available to consumers. While the results of this study offer valuable insights into how respondents sought and processed information about the product, it is not unlikely that public responses may differ once these products become available for purchase and consumption. Additionally, the study provided only a limited explanation of CRISPR, which may have influenced the responses of participants whose only exposure to the technology came from the information presented in the study. Finally, although we used quota sampling to reflect U.S. Census distributions, the use of a non-probability online panel may limit the generalizability of the results to the broader U.S. population.

## Conclusions

This study contributes to the growing body of research on public engagement with emerging food technologies by examining how consumers seek, process, and avoid information about CRISPR-edited pork products. Consistent with the RISP model, information sufficiency and information sufficiency thresholds were important drivers of information-seeking and avoidance behaviors. Information seeking was strongly associated with systematic processing, suggesting that consumers who actively seek information are likely to evaluate information carefully and critically. The strong interrelationships among different communication channels further suggest that consumers use multiple sources of information rather than relying on a single channel. Respondents with some college education reported higher levels of information seeking, while older adults and individuals with higher education levels were less likely to avoid information. These results suggest that demographic characteristics may play a supporting role in shaping information behaviors and should be considered alongside psychographic factors when developing communication strategies. This study also found that consumers are generally open to learning about CRISPR-edited pork products and possess high information sufficiency threshold. These findings have several practical implications. First, communicators should provide detailed, evidence-based information about CRISPR-edited pork products, as consumers appear willing to engage with substantive content rather than simplified messages. Second, communication efforts should employ coordinated, multi-channel strategies that integrate social media, news media, Extension, and interpersonal communication to maximize reach and reinforce message consistency. Third, audience segmentation should prioritize psychographic characteristics such as values, beliefs, trust, and information needs while also accounting for key demographic differences in information engagement. Finally, Extension organizations should be equipped with accurate and up-to-date information on gene-editing technologies to support informed public engagement. These strategies can help encourage transparency, trust, and informed decision-making as CRISPR-edited food products enter the marketplace.

## Data Availability

Data can be made available on request.
